# Vascular habitat analysis using dynamic susceptibility contrast-enhanced perfusion MRI for differentiating glioblastoma from solitary brain metastases

**DOI:** 10.3389/fonc.2026.1904498

**Published:** 2026-10-07

**Authors:** Xuan Li, Hanwei Wang, Linlan Zeng, Mimi Zhao, Ling Feng, Huan Xie, Qisheng Ran, Qiang Ma, Shunan Wang

**Affiliations:** 1 Department of Radiology, Daping Hospital, Army Medical University, Chongqing, China; 2 Chongqing Clinical Research Center of Imaging and Nuclear Medicine, Chongqing, China; 3 Department of Pathology, Daping Hospital, Army Medical University, Chongqing, China

**Keywords:** brain metastases, glioblastoma, habitat imaging, magnetic resonance imaging, perfusion

## Abstract

**Objective:**

To investigate tumor vascular habitat parameters based on dynamic susceptibility contrast-enhanced perfusion-weighted imaging (DSC-PWI) for differentiating glioblastoma (GBM) from solitary brain metastasis (SBM).

**Methods:**

In this single-center retrospective study, imaging data from 141 patients with pathologically or clinically confirmed malignant brain tumors (74 GBM, 67 SBM) were analyzed. The hemodynamic tissue signature (HTS) method was used to segment the lesions and construct four tumor vascular habitats: high angiogenic tumor (HAT), low angiogenic tumor (LAT), potentially infiltrated peripheral edema (IPE), and vasogenic peripheral edema (VPE). Pearson correlation analysis was used to quantify the correlations among habitat hemodynamic parameters. Habitat characteristic parameters were calculated, and ROC were plotted to evaluate the diagnostic performance. A nomogram model was constructed using logistic regression.

**Results:**

GBM exhibited significantly higher median relative cerebral blood volume (rCBV_median_) and median relative cerebral blood flow (rCBF_median_) than SBM across all four habitat regions (all *p* < 0.001). In terms of volumetric parameters, the ratio of IPE volume to peritumoral volume (*V*_(IPE)_/*V*_(P)_) was significantly higher in the GBM group (*p* < 0.001). Among individual habitat parameters, LAT rCBF_median_ showed the best diagnostic performance with an AUC of 0.786 (95% CI: 0.709-0.850). In the GBM group, the perfusion parameters of the IPE and VPE were significantly positively correlated with those of the HAT, whereas no such correlation was found in the SBM group. A nomogram incorporating lesion location, enhancement pattern, and LAT-rCBF_median_ yielded an AUC of 0.857 (95% CI: 0.79-0.92) for distinguishing GBM from SBM, with good calibration.

**Conclusion:**

Tumor vascular habitat parameters based on DSC-PWI demonstrate significant differences between newly diagnosed GBM and SBM, and the nomogram shows certain value in differentiating between the two tumor types.

## 1 Introduction

Gliomas and brain metastases represent the most prevalent malignant intracranial neoplasms in adults. Glioma accounts for approximately 22.2% of all malignant primary central nervous system tumors, with glioblastoma (GBM) being the most common, comprising approximately 57.3% of gliomas (1). In comparison, brain metastases are nearly 10 times more common than GBM (2).

Both GBM and solitary brain metastases (SBM) are highly aggressive brain tumors with poor prognoses (3). However, their treatment strategies differ significantly: newly diagnosed GBM is typically managed with maximal safe resection followed by adjuvant chemoradiotherapy (4), while brain metastases require comprehensive treatment strategy that may include surgical resection, whole-brain radiotherapy, systemic chemotherapy, and targeted therapies (5). Consequently, accurate preoperative differentiation is crucial. Although histopathological examination remains the diagnostic gold standard, its invasive nature limits its applicability, making non-invasive imaging essential. MRI is the most important imaging modality for intracranial tumors. On conventional MRI, GBM typically presents as a large, irregular lesion in the deep white matter with heterogeneous rim enhancement, whereas SBM often presents as a smaller, uniformly enhancing lesion in the superficial white matter (6, 7). However, both tumors can exhibit overlapping imaging features such as rim enhancement, extensive peritumoral edema, cystic changes, necrosis, and hemorrhage (8). Particularly in SBM cases without a known primary tumor, accurate differentiation becomes exceedingly difficult (9, 10). Therefore, precise non-invasive preoperative differentiation between GBM and SBM is both critically important for treatment decisions and highly challenging.

Advanced imaging techniques, including diffusion-weighted imaging, perfusion-weighted imaging, and magnetic resonance spectroscopy, have been used to differentiate these tumors, however, their clinical utility remains suboptimal. As GBM and SBM are biologically distinct entities with differing angiogenic and hemodynamic profiles, habitat imaging holds considerable promise for enhancing their differentiation through the visualization and quantification of microenvironmental heterogeneity, thereby offering a novel avenue for precision imaging in the preoperative setting (11). Habitat imaging holds promise for differentiating GBM and SBM (two biologically distinct tumors) by visualizing and quantifying heterogeneity, providing a novel approach for preoperative precision imaging.

In this study, we constructed DSC-PWI-based tumor vascular habitats to visualize the spatial heterogeneity of the tumor vascular microenvironment, quantitatively analyzed hemodynamic and volumetric habitat features, and developed a clinical diagnostic model to evaluate the utility of this imaging technique in the preoperative differential diagnosis of GBM and SBM.

## 2 Materials and methods

### 2.1 Study patient

This retrospective study was approved by the Institutional Review Board of the Army Medical Center of the People’s Liberation Army of China, which granted a waiver of informed consent. We retrospectively reviewed clinical and imaging data of 141 patients with pathologically or clinically confirmed GBM or SBM who were admitted to our center between January 2019 and March 2025.Inclusion criteria were as follows: (1) postoperative pathologically confirmed GBM; (2) postoperative pathologically or clinically confirmed SBM; (3) brain MRI performed within one week before treatment. Exclusion criteria were as follows: (1) recurrent tumors; (2) coexistence of other intracranial pathology; (3) age under 18 years; (4) history of cranial radiotherapy or chemotherapy before MRI; (5) incomplete MRI sequences or poor image quality with significant artifacts. Finally, 74 GBM patients and 67 SBM patients satisfied the eligibility criteria. All GBM cases were confirmed as IDH-wildtype according to the 2021 World Health Organization (WHO) classification of tumors of the central nervous system. IDH status was determined by immunohistochemistry. The final GBM cohort consisted of 74 IDH-wildtype glioblastoma patients. Among the 67 SBM patients, 41 were pathologically confirmed (by surgical resection or biopsy) and 26 were clinically confirmed (based on imaging findings and history of primary malignancy). For the SBM cases, the primary tumors included lung cancer (n = 52), gastrointestinal tumors (n = 10), ovarian cancer (n = 3), and breast cancer (n = 2). The patient inclusion flowchart is shown in **Figure 1**.

**Figure 1:**
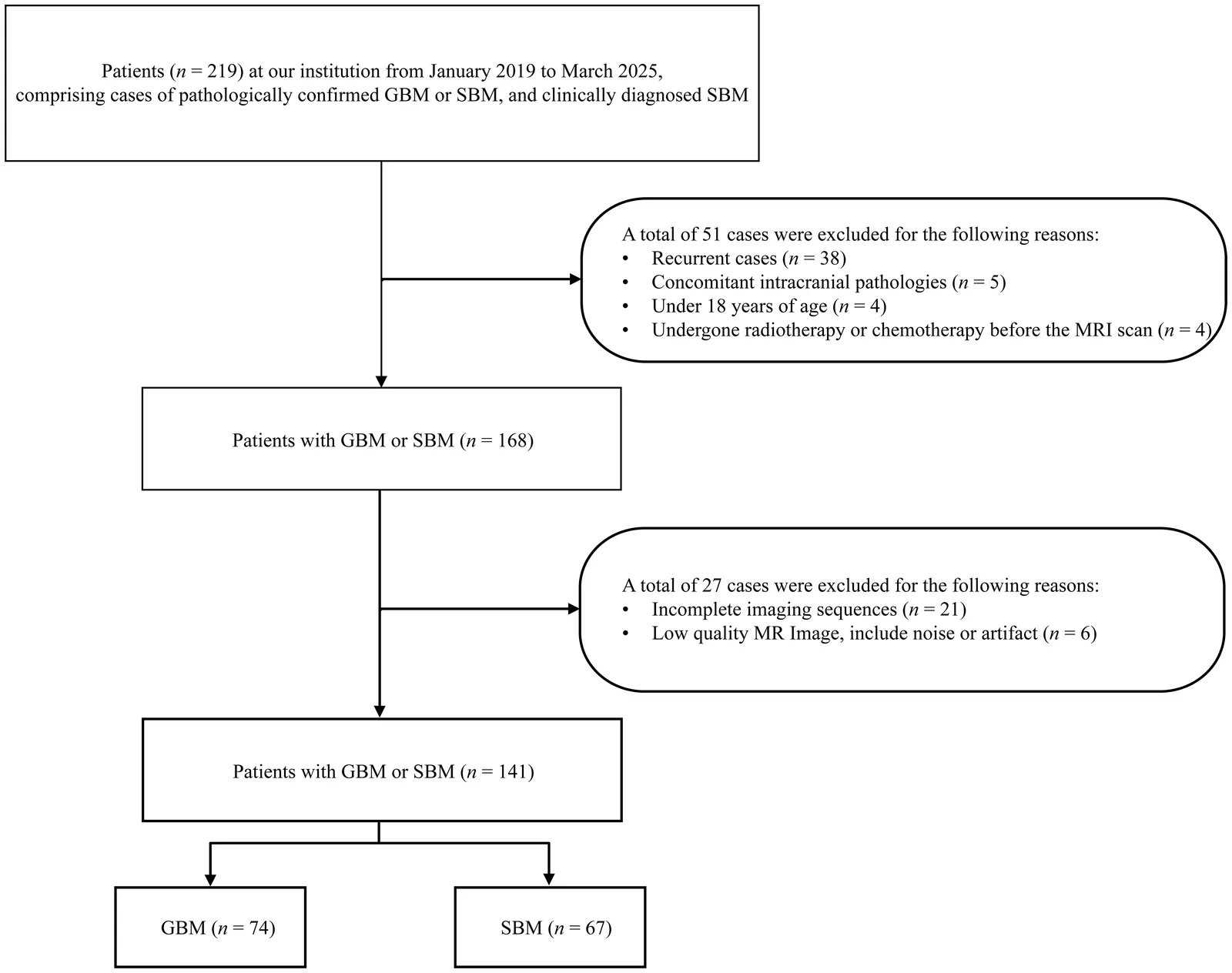
Clinical case inclusion and exclusion flowchart.

### 2.2 Image acquisition

All patients were scanned using a Siemens Verio 3.0 T superconducting MRI scanner with an 8-channel head coil. MRI sequences included T1WI, T2WI, FLAIR, CE-T1WI, and DSC-PWI. Detailed scanning parameters are provided in **Supplementary Table 1**. For both CE-T1WI and DSC-PWI, the contrast agent gadopentetate dimeglumine (Beijing Beilu Pharmaceutical Co., Ltd.) was administered via intravenous bolus injection at a dose of 0.2 mL/kg, with flow rates of 3 mL/s and 4 mL/s, respectively.

### 2.3 Image processing

Volumes of interest (VOI) were manually delineated using ITK-SNAP 4.2, including the tumor core (the enhancing rim and internal components, including cystic, necrotic, and hemorrhagic components on CE-T1WI) and peritumoral region (non-enhancing FLAIR-hyperintense area surrounding the tumor core).

Tumor vascular habitats, namely the high angiogenic tumor (HAT), low angiogenic tumor (LAT), potentially infiltrated peripheral edema (IPE), and vasogenic peripheral edema (VPE), were constructed using the ONCOhabitats (www.oncohabitats.upv.es) online platform. Using this service requires importing T1WI, CE-T1WI, T2WI, T2-FLAIR, and raw DSC-PWI images.

The method includes the following four stages: (1) Image preprocessing: comprising correction of common MRI artifacts (such as magnetic field inhomogeneities and noise), multimodal registration, brain extraction, and motion correction. (2) Tumor segmentation: which employs a deep learning 3D convolutional neural network model to automatically segment enhancing tumor, edema, and necrotic tissue. The core of this model is based on the Directional Class Adaptive Spatially Varying Finite Mixture Model (DCA-SVFMM). (3) DSC perfusion quantification: Contrast agent leakage is corrected using the Boxerman method, followed by gamma-variate curve fitting. Relative cerebral blood volume (rCBV) is calculated by numerical integration of the area under the gamma-variate curve, while relative cerebral blood flow (rCBF) is calculated using the block-circulant singular value decomposition deconvolution algorithm. Both rCBV and rCBF maps are normalized to the contralateral normal-appearing white matter. (4) the HTS habitats were delineated: a two-stage DCA-SVFMM clustering of the rCBV and rCBF maps was performed—initially, the entire enhancing tumor and edema region was separated into enhancing-tumor-at-DSC (ETDSC) and edema-at-DSC (EDDSC) classes; subsequently, within each class, a second clustering step partitioned ETDSC into HAT and LAT, and EDDSC into IPE and VPE. To ensure reproducibility, the clustering was deterministically initialized, and several anatomical constraints (e.g., distance to enhancing margin, minimum habitat size) were applied to avoid misclassification. The resulting habitat maps were visually inspected for quality and overlaid on anatomical images. The robustness of the HTS method for processing MR images and calculating rCBV across different hospitals and acquisition parameters has been validated in previous multicenter studies.A more detailed description of this method is provided in (12). The robustness of the HTS method for processing MR images and calculating rCBV across different hospitals and acquisition parameters has been validated in previous multicenter studies (11). The vascular habitat processing workflow is shown in **Figure 2**.

**Figure 2:**
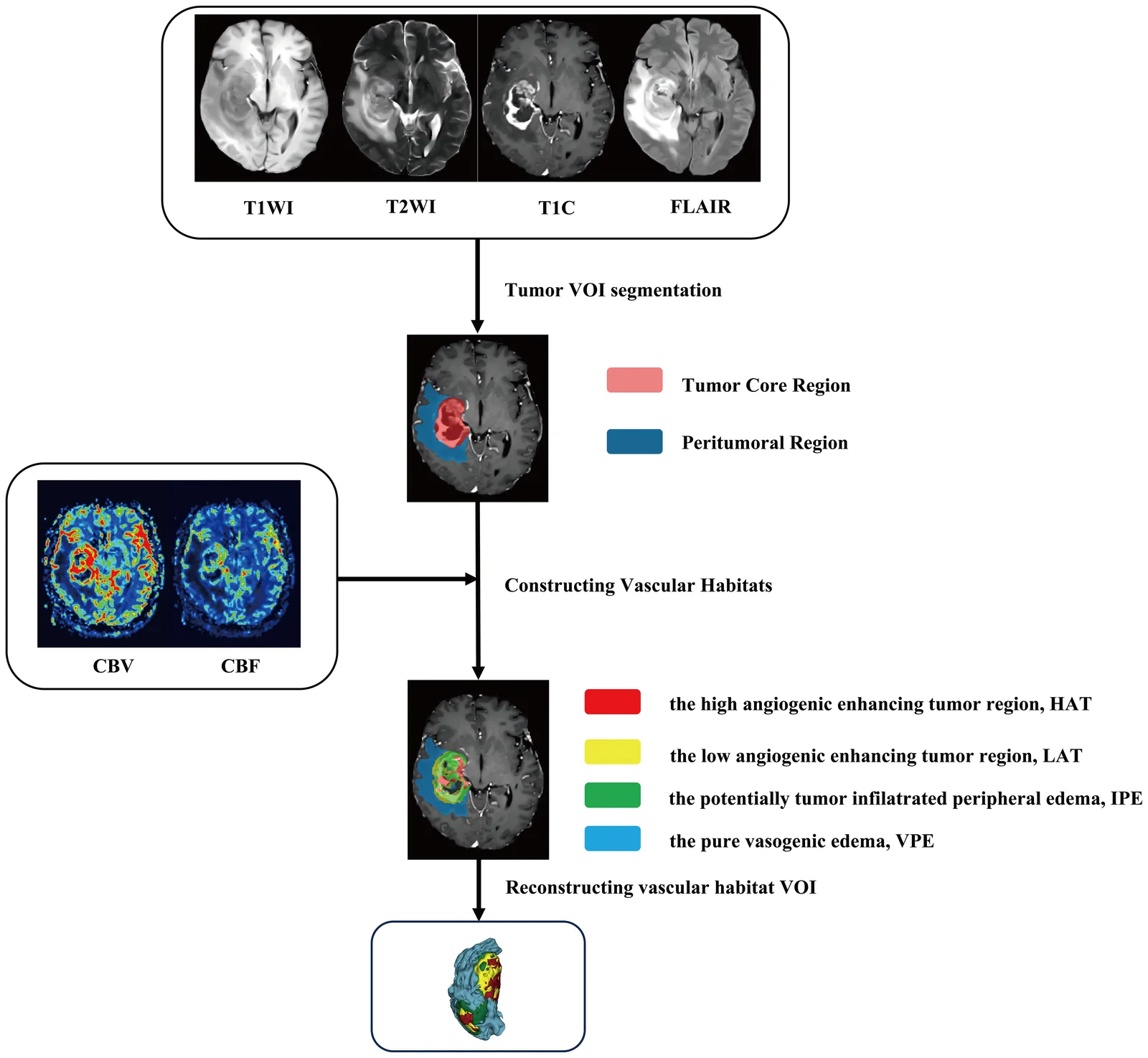
Vascular habitat processing flowchart.

### 2.4 Quality control and data collection

Two neuroradiologists (with 10 and 13 years of experience in neuroradiology, respectively), blinded to clinical data, independently performed MRI feature analysis and tumor segmentation. Volumes of the tumor core (*V*_(T)_), peritumoral edema (*V*_(P)_), and the volumes of each habitat were calculated. Discrepancies were resolved via consensus discussion. Interobserver agreement was assessed using Kappa statistics, with Kappa values of 0.75-1.00 interpreted excellent agreement.

Conventional imaging features assessed included enhancement pattern, peritumoral edema, intratumoral hemorrhage, necrosis, and midline shift. Lesion locations were determined based on preoperative MRI and classified into frontal lobe, temporal lobe, parietal lobe, occipital lobe, and other locations. Other locations were defined as tumors with the main lesion situated in the basal ganglia, thalamus, brainstem, cerebellum, or periventricular region, without clear attribution to a specific cerebral lobe. When a tumor involved two or more adjacent lobes, the lobe in which the lesion occupied the largest volume percentage was assigned as the final location based on volumetric assessment. Enhancement patterns were categorized as ring enhancement and patchy enhancement. Ring enhancement was defined as a heterogeneous, closed enhancing rim surrounding a central non-enhancing region on CE-T1WI. Patchy enhancement was defined as irregular, patchy or geographic areas of enhancement on CE-T1WI. Habitat characteristics included volumetric parameters (ratio of each habitat volume to *V*_(T)_ or *V*_(P)_) and hemodynamic parameters (rCBV and rCBF of four habitats), calculated using numerical integration of the area under the gamma-variate curve and the block-circulant singular value decomposition deconvolution scheme.

### 2.5 Statistical analysis

Statistical analyses were performed using SPSS 27.0, MedCalc 20.100, and R 4.4.2 software. Normally distributed continuous variables were presented as mean ± standard deviation, non-normally distributed variables as median with interquartile range (*P*_25_, *P*_75_), and categorical data as frequency (*n*) and percentage (%). Between-group comparisons were conducted using independent-sample *t*-test or Mann-Whitney *U* test for continuous variables, and *χ^2^* test or Fisher’s exact test for categorical data.

The diagnostic performance of habitat parameters was evaluated using ROC curve analysis, with AUC, sensitivity, and specificity calculated. Variables with *p* < 0.05 in univariate logistic regression were entered into multivariate logistic regression to construct a nomogram model, whose performance was evaluated via ROC curves, calibration curves, and 1000-bootstrap repeated validation. Pearson correlation analysis was used to assess correlations between habitat hemodynamic parameters. All statistical tests were two-sided, and *p* < 0.05 was considered statistically significant.

## 3 Results

### 3.1 Baseline clinical and imaging characteristics

The GBM group included 74 patients (45 males, 29 females; mean age 55.84 ± 12.62 years), and the SBM group included 67 patients (35 males, 32 females; mean age 58.03 ± 7.34 years). Interobserver agreement was excellent for all imaging features (Kappa = 0.825-1.000, *p* < 0.001). No significant between-group differences were observed with respect to age, sex, peritumoral edema, intratumoral hemorrhage, necrosis, or midline shift (all *p* > 0.05). In contrast, statistically significant differences were observed in lesion location (*p* < 0.001) and enhancement pattern (*p* = 0.001), as detailed in **Table 1**.

**Table 1:** Comparison of baseline clinical and imaging characteristics of the patients [*n* (%), ( $\bar{x}$ ± s)].

Clinical data	GBM (*n* = 74)	SBM (*n* = 67)	*t/χ^2^/Z*	*p*
Age (years)	55.84 ± 12.62	58.03 ± 7.34		0.324
Gender			1.053	0.305
Male	45(60.81)	35(52.24)		
Female	29(39.19)	32(47.76)		
Lesion Location			26.464	<0.001
Frontal Lobe	21(28.38)	8(11.94)		
Temporal Lobe	23(31.08)	8(11.94)		
Parietal Lobe	16(21.62)	18(26.87)		
Occipital Lobe	3(4.05)	21(31.34)		
Other Locations	11(14.87)	12(17.91)		
Enhancement Pattern			10.612	0.001
Ring-shaped Enhancement	57(77.03)	34(50.75)		
Patchy Enhancement	17(22.97)	33(49.25)		
Peritumoral Edema			0.010	1.000
Yes	72(97.30)	65(97.01)		
No	2(2.70)	2(2.99)		
Hemorrhage			2.716	0.099
Yes	66(89.19)	53(79.10)		
No	8(10.81)	14(20.90)		
Necrosis			0.535	0.465
Yes	66(89.19)	57(85.07)		
No	8(10.81)	10(14.93)		
Midline Shift			0.594	0.441
Yes	52(70.27)	43(64.18)		
No	22(29.73)	24(35.82)		

Other locations were defined as tumors with the main lesion situated in the basal ganglia, thalamus, brainstem, cerebellum, or periventricular region, without clear attribution to a specific cerebral lobe.

### 3.2 Between-group comparison of habitat parameters

For volumetric parameters, *V*_(IPE)_/*V*_(P)_ was significantly higher in the GBM group than in the SBM group (*p* < 0.001), whereas *V*_(HAT)_/*V*_(T)_, *V*_(LAT)_/*V*_(T)_, and *V*_(VPE)_/*V*_(P)_ showed no significant between-group differences (*p* = 0.172, 0.162, and 0.265, respectively) (**Figure 3**; **Table 2**). For hemodynamic parameters, rCBV_median_ and rCBF_median_ in all four habitats (HAT, LAT, IPE, VPE) were significantly higher in the GBM group than in the SBM group (all *p* < 0.001) (**Figure 4**; **Table 2**).

**Figure 3:**
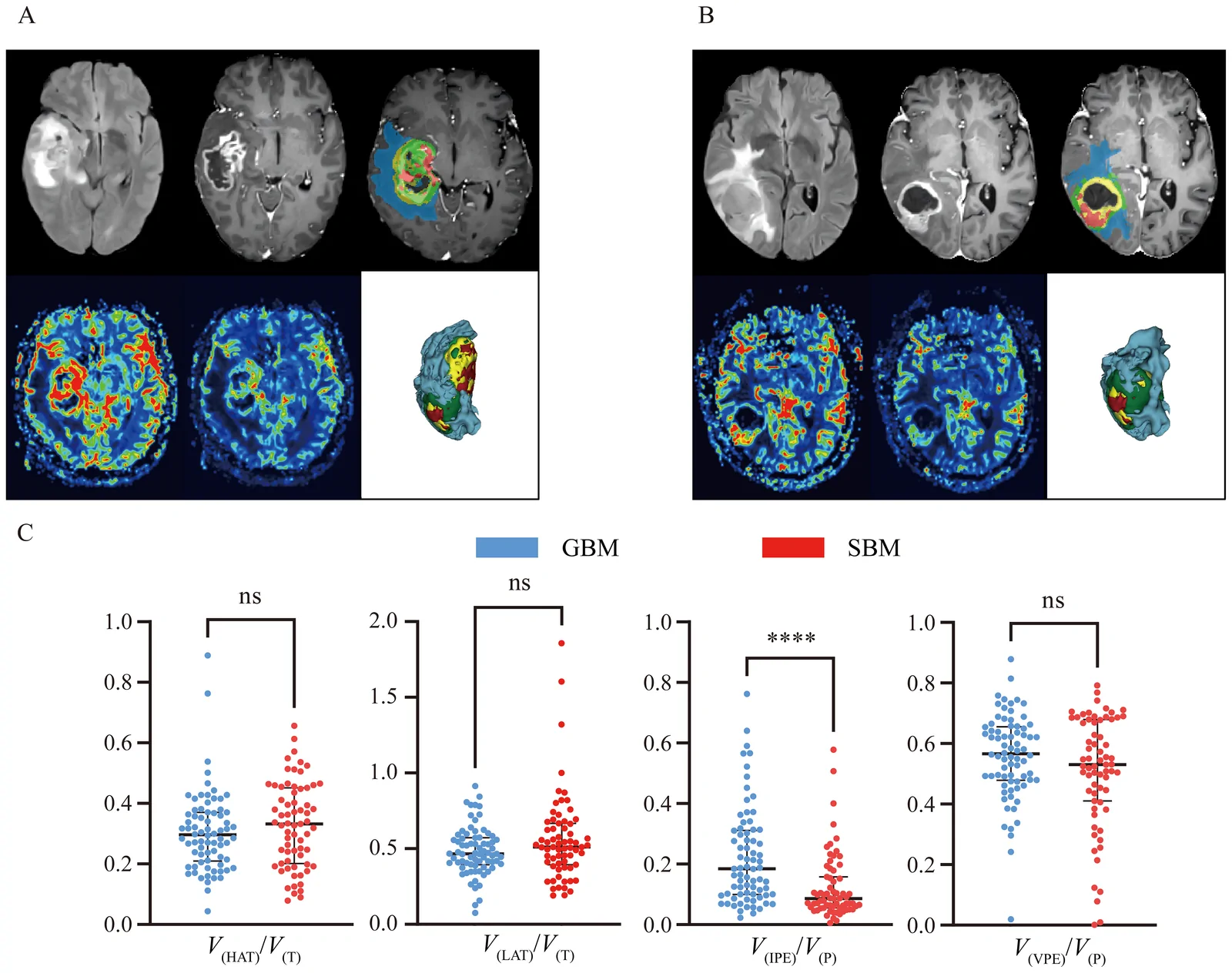
Conventional imaging, habitat segmentation, and comparison of habitat volume parameters between GBM and SBM. **(A)** A 56-year-old male patient with GBM. **(B)** A 55-year-old male patient with brain metastasis from lung cancer. For each, images show CE-T1WI, FLAIR, CBV, CBF, habitat maps (four colors for four habitats), and 3D renderings. **(C)** Scatter plot comparing habitat volume ratios between groups. **** indicates p < 0.0001.

**Table 2:** Comparison of habitat characteristic parameters in GBM and SBM groups.

Parameter	GBM (*n* = 74)	SBM (*n* = 67)	*p*
*V*_(HAT)_/*V*_(T)_	0.305 ± 0.133	0.329 ± 0.140	0.172
*V*_(LAT)_/*V*_(T)_	0.467 (0.394-0.573)	0.512 (0.393-0.667)	0.162
*V*_(IPE)_/*V*_(P)_	0.184 (0.099-0.311)	0.086 (0.055-0.158)	<0.001
*V*_(VPE)_/*V*_(P)_	0.567 (0.478-0.655)	0.530 (0.411-0.679)	0.265
HAT-rCBV_median_	5.967 ± 1.788	4.613 ± 1.844	<0.001
LAT-rCBV_median_	3.124 (2.409-3.911)	2.271 (1.819-2.660)	<0.001
IPE-rCBV_median_	2.037 (1.608-2.717)	1.572 (1.094-2.006)	<0.001
VPE-rCBV_median_	0.832 (0.682-1.102)	0.640 (0.508-0.783)	<0.001
HAT-rCBF_median_	4.451 ± 1.451	3.785 ± 1.207	<0.001
LAT-rCBF_median_	2.427 (2.034-3.028)	1.878 (1.514-2.311)	<0.001
IPE-rCBF_median_	1.537 (1.210-2.145)	1.186 (0.891-1.527)	<0.001
VPE-rCBF_median_	0.753 (0.652-1.023)	0.608 (0.490-0.752)	<0.001

Data are presented as mean ± SD for normally distributed variables, and as median (interquartile range) for non-normally distributed variables.

**Figure 4:**
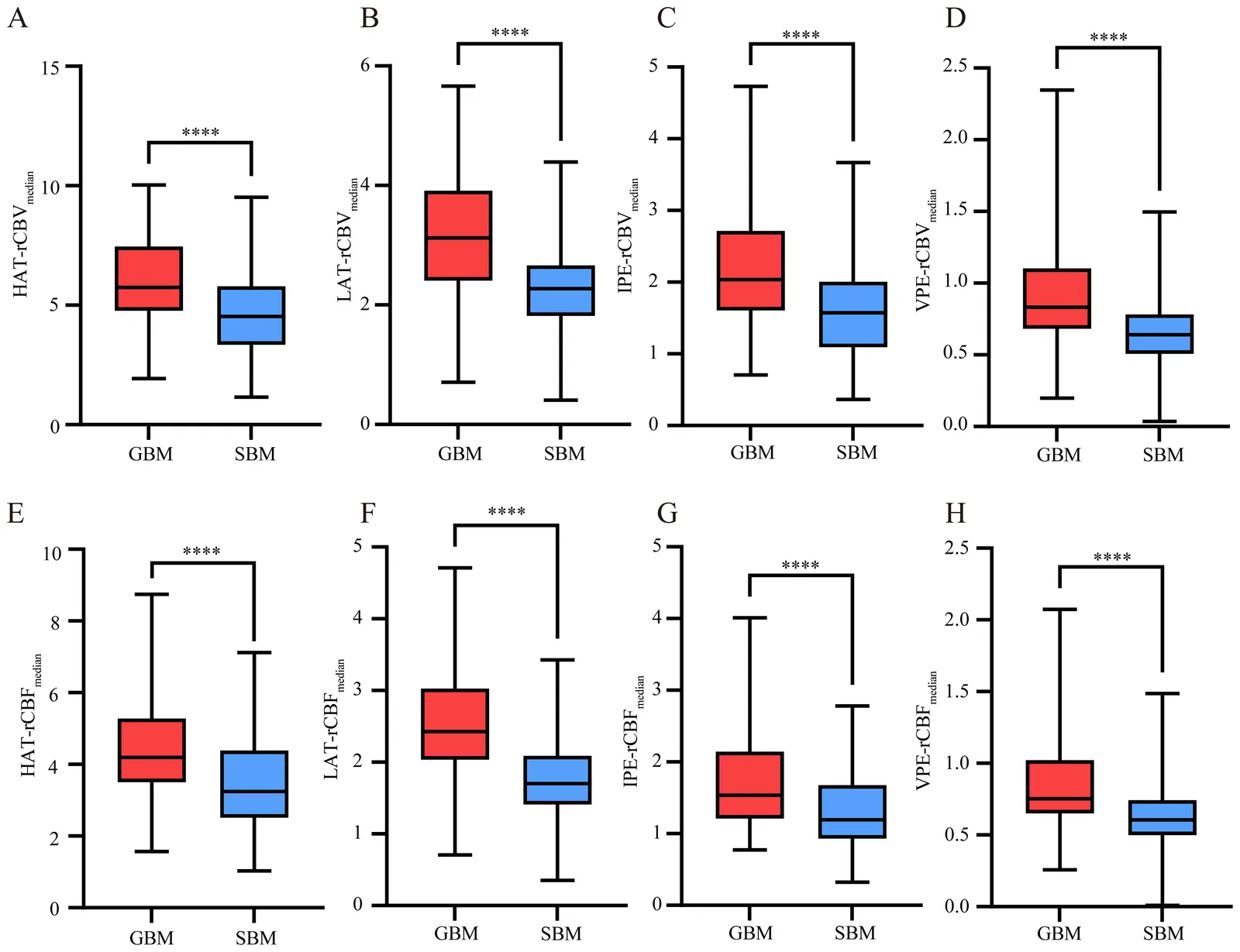
Comparison of hemodynamic parameters between GBM and SBM across different habitats. **(A–D)** Comparison of rCBV_median_ in the HAT, LAT, IPE, and VPE, respectively. **(E–H)** Comparison of rCBF_median_ in the HAT, LAT, IPE, and VPE, respectively. **** indicates p < 0.0001.

### 3.3 Diagnostic efficacy of habitat characteristics

The ROC curve analysis was performed for the volume parameters and hemodynamic parameters of the four habitats (**Figure 5**; **Table 3**).

**Figure 5:**
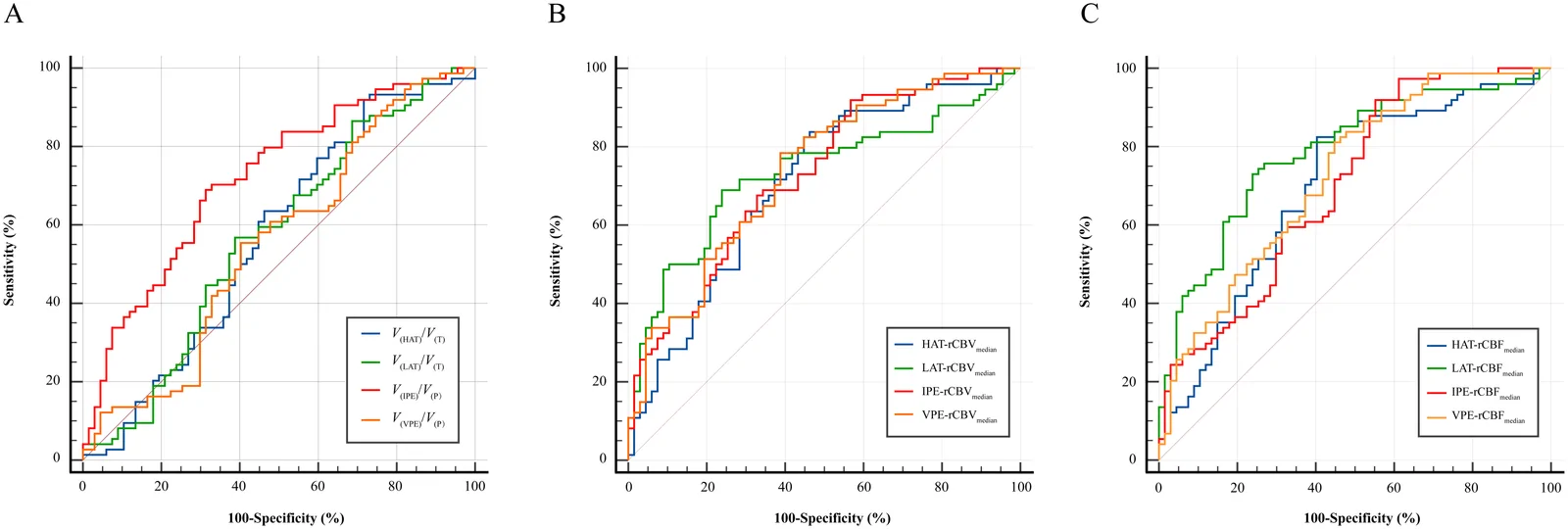
**(A-C)** Receiver operating characteristic curves of the volume parameters, rCBV_median_ and rCBF_median_ for the four habitats in differentiating GBM from SBM, respectively.

**Table 3:** Diagnostic efficacy of characteristic parameters of various habitats.

Factors	AUC (95%*CI*)	Sensitivity (%)	Specificity (%)	Cut-off value
*V*_(HAT)_/*V*_(T)_	0.567(0.481-0.650)	91.89	28.36	0.428
*V*_(LAT)_/*V*_(T)_	0.568(0.482-0.651)	59.76	61.19	0.484
*V*_(IPE)_/*V*_(P)_	0.726(0.638-0.793)	68.92	68.66	0.109
*V*_(VPE)_/*V*_(P)_	0.554(0.468-0.638)	55.41	59.70	0.556
HAT-rCBV_median_	0.708(0.626-0.782)	82.43	55.22	4.635
LAT-rCBV_median_	0.732(0.651-0.803)	68.92	76.12	2.660
IPE-rCBV_median_	0.727(0.646-0.799)	91.89	43.28	1.331
VPE-rCBV_median_	0.735(0.654-0.806)	78.38	61.19	0.677
HAT-rCBF_median_	0.699(0.616-0.773)	82.43	59.70	3.392
LAT-rCBF_median_	0.786(0.709-0.850)	72.97	76.12	2.092
IPE-rCBF_median_	0.701(0.618-0.775)	91.89	44.78	1.052
VPE-rCBF_median_	0.725(0.643-0.797)	81.08	55.22	0.635

ROC curve analysis showed that *V*_(IPE)_/*V*_(P)_ achieved an AUC of 0.726 (95%CI: 0.638-0.793) for differentiating GBM from SBM. Among all hemodynamic parameters, LAT-rCBF_median_ exhibited the highest single-parameter diagnostic performance, with an AUC of 0.786 (95% CI: 0.709-0.850), a sensitivity of 72.97%, and a specificity of 76.12% at the optimal cutoff value of 2.092.

### 3.4 Correlation analysis of habitat parameters

Pearson correlation analysis was performed to examine the relationships among hemodynamic parameters across the four vascular habitats, A cross-habitat correlation matrix was computed for the GBM and SBM groups (**Figure 6**). The results showed that HAT-rCBV_median_ was significantly positively correlated with IPE-rCBV_median_ (*r* = 0.574, *p* < 0.01) and VPE-rCBV_median_ (r = 0.244, *p* < 0.05) in the GBM group. In contrast, no such correlations were observed within the SBM group. Consistent results were found for rCBF_median_, as detailed in **Supplementary Table 2**.

**Figure 6:**
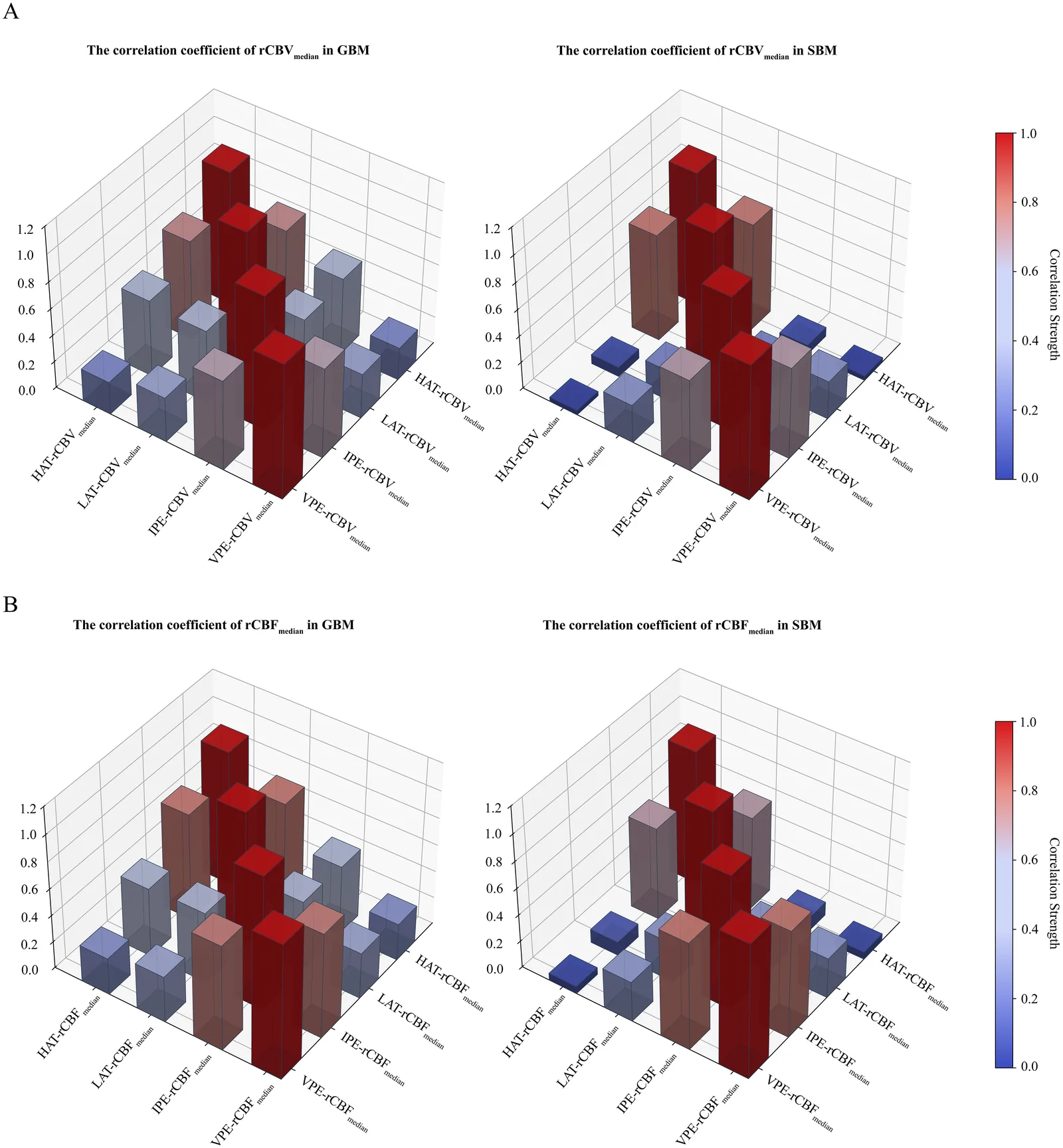
Comparison of habitat region interactions between the GBM and SBM groups. **(A)** Habitat region interactions of rCBV_median_. **(B)** Habitat region interactions of rCBF_median_. In the GBM group, HAT-rCBV_median_ showed significant positive correlations with IPE-rCBV_median_ and VPE-rCBV_median_, while in the SBM group, HAT-rCBV_median_ exhibited no significant correlations with IPE-rCBV_median_ or VPE-rCBV_median_. Similar findings were observed for rCBF_median_.

### 3.5 Performance of the predictive nomogram

Multivariate logistic regression analysis identified occipital lobe location (OR = 0.079 95%CI: 0.013-0.479, *p* < 0.01), enhancement pattern (OR = 0.177, 95%CI: 0.064-0.495, *p* = 0.001), and LAT-rCBF_median_ (OR = 8.210, 95%CI: 1.511-44.623, *p* = 0.015) as independent differentiating factors for GBM. A nomogram model constructed incorporating these factors achieved an AUC of 0.857 (95%CI: 0.79-0.92). The model demonstrated favorable calibration, as evidenced by the calibration curve and a non-significant Hosmer–Lemeshow test (*p* = 0.739) (**Figures 7**, **8**).

**Figure 7:**
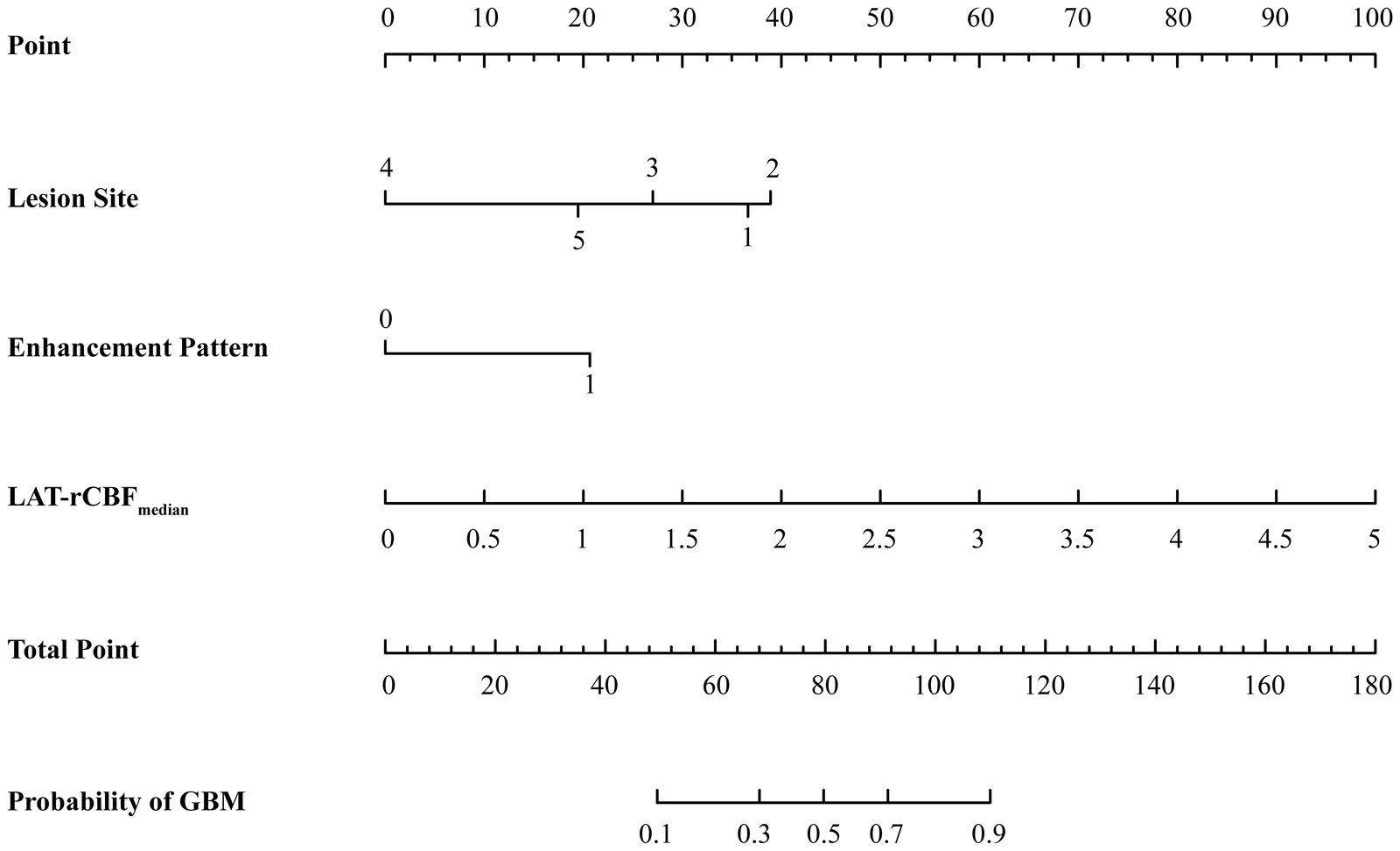
A nomogram incorporating enhancement pattern, lesion site, and LAT-rCBF_median_. Lesion site was coded as: 1 = frontal lobe, 2 = temporal lobe, 3 = parietal lobe, 4 = occipital lobe, 5 = other locations. Enhancement pattern was coded as: 0 = patchy enhancement, 1 = ring enhancement. Points are assigned to each variable, and the total points correspond to the predicted probability of GBM.

**Figure 8:**
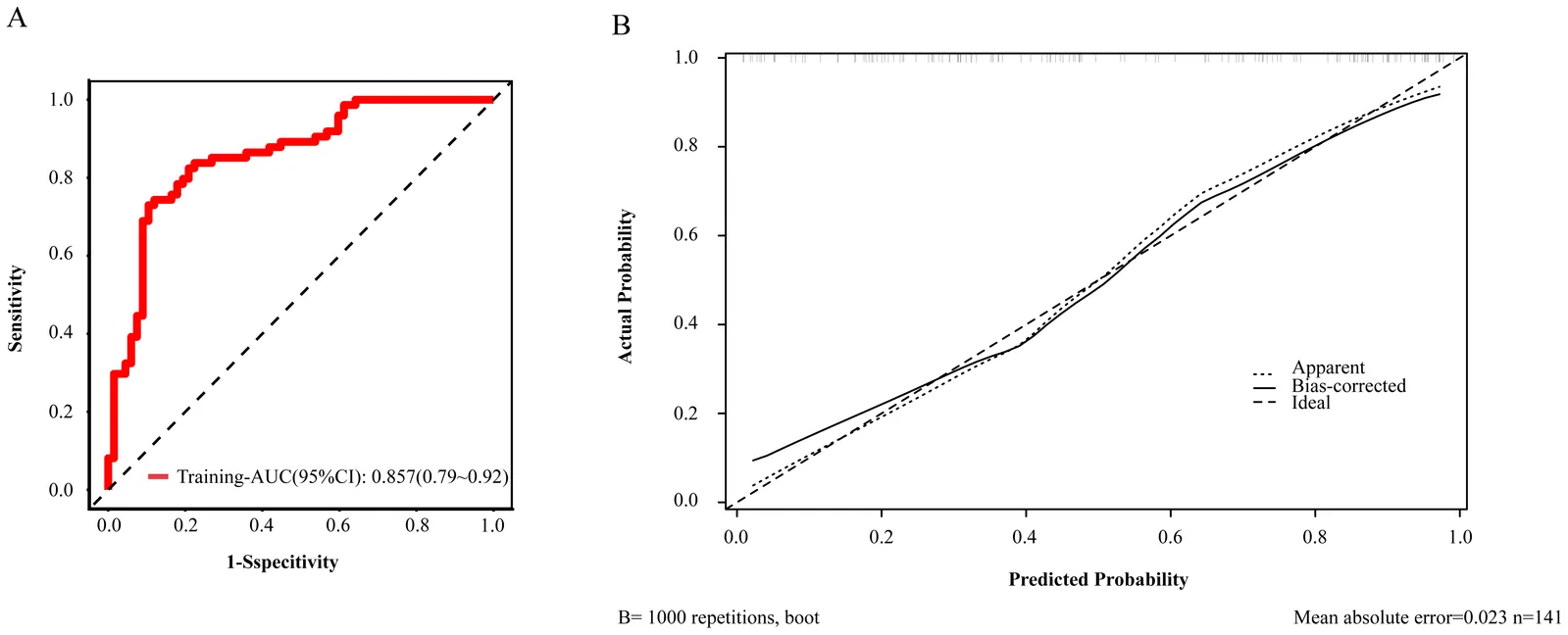
**(A)** Receiver operating characteristic (ROC) curve of the nomogram. The area under the curve (AUC) is 0.857 (95% CI: 0.79-0.92). **(B)** Calibration curve of the nomogram. The x-axis represents the predicted probability, and the y-axis represents the actual probability. The dashed diagonal line indicates ideal calibration. The solid line shows the bias-corrected calibration, with a mean absolute error of 0.039, indicating good agreement between predicted and observed probabilities.

## 4 Discussion

In this study, we used DSC-PWI to construct tumor vascular habitats and assessed the value of habitat characteristic parameters in the discrimination of GBM and SBM. The results indicate that the volume parameter *V*_(IPE)_/*V*_(P)_ and hemodynamic parameters (rCBV_median_, rCBF_median_) obtained from tumor vascular habitats based on DSC-PWI can serve as quantitative imaging markers to aid preoperative differential diagnosis.

A key feature of high-grade gliomas is the expansion of the peritumoral edema region, which reflects extensive tumor infiltration (13, 14). Radiomic features of this region can predict glioma recurrence, and rCBV in the IPE serves as an imaging biomarker for predicting local recurrence patterns (15). To a considerable extent, the IPE region reflects the heterogeneity of the peritumoral edema area and represents the region of infiltrative tumor growth. In this study, *V*_(IPE)_/*V*_(P)_ was significantly higher in GBM than in SBM, indicating that infiltrative growth of GBM tumor cells is more active and extensive. Therefore, *V*_(IPE)_/*V*_(P)_ not only plays an important role in predicting glioma recurrence but can also serve as an indicator for differentiating GBM from SBM.

Askaner et al. found no significant difference in rCBV between GBM and SBM within the contrast-enhancing tumor parenchyma on conventional DSC images, whereas rCBV in the peritumoral edema was significantly higher in glioblastoma (16). Similarly, Li et al. and Trinh et al. reported that intratumoral rCBV derived from conventional DSC could not distinguish the two tumors, whereas peritumoral rCBV showed good discriminative performance (17, 18). These studies consistently indicate that in the tumor parenchyma, DSC-derived perfusion parameters have limited value for differentiating glioblastoma from solitary brain metastasis, and all of these investigations relied on conventional ROI-based measurements, treating the tumor core or peritumoral region as a single homogeneous compartment. However, in our study, tumor vascular habitat analysis revealed that hemodynamic parameters in all four habitat regions of GBM were higher than those in SBM, all demonstrating good diagnostic performance. Furthermore, previous investigations have indicated that rCBV reflects the degree of microvascular proliferation within the tumor, and increased microvascular structure and neovascularization can lead to elevated rCBV values (19–21). Our findings are consistent with these reports, which may be attributed to differences in the vascular structure, distribution, and functional status between GBM and SBM. Compared with previous studies, the habitat analysis method adopted in this study automatically segmented the entire lesion and peritumoral region into four distinct habitat subregions. By capturing the spatial heterogeneity of the tumor vascular microenvironment, this subregional analysis overcomes the averaging effect inherent in conventional ROI methods, thereby providing more comprehensive information.

Tumor cells migrate from the core to the peritumoral region, and expansion of the peritumoral edema region is closely associated with invasive tumor behavior (13, 22). In our correlation analysis, IPE-rCBV_median_ and VPE-rCBV_median_ in GBM showed significant correlations with HAT-rCBV_median_, whereas no such correlations were observed in SBM. Similar results were found for rCBF_median_. These findings indicate that heterogeneity of the GBM tumor parenchyma is closely associated with the peritumoral edema region and, consistent with previous reports (13, 22). The potential of the observed differences in correlations to serve as auxiliary indicators for differentiating these two tumor types remains to be confirmed by formal statistical comparison.

Hemodynamic parameters from all four habitat regions demonstrated the ability to differentiate GBM from SBM. Among all parameters, LAT-rCBF_median_ exhibited the best diagnostic performance with an AUC of 0.786. Additionally, *V*_(IPE)_/*V*_(P)_ was higher in GBM than in SBM. These findings suggest significant differences in vascular heterogeneity within the IPE region between the two tumor types, reflecting distinct mechanisms of tumor vascular leakage, peritumoral edema formation, and microenvironmental regulation in GBM and SBM, thereby providing new insights for clinical differential diagnosis. A predictive model was constructed integrating clinical baseline characteristics with habitat parameters to enhance the accuracy of preoperative discrimination between GBM and SBM. This model incorporated morphological features such as tumor location and enhancement pattern, as well as LAT-rCBF_median_, an imaging biomarker representing tumor vascular heterogeneity. The model achieved an AUC of 0.857 with good calibration, indicating excellent discriminative ability. While previous studies have mostly relied on single parameters such as rCBV or radiomic features for differentiation, this study incorporated habitat characteristic parameters, placing greater emphasis on the quantification of the tumor microenvironment.

Currently, techniques such as mean apparent propagator-MRI (MAP-MRI) and neurite orientation dispersion and density imaging (NODDI) have been employed to differentiate GBM from SBM, but results have been suboptimal (23–25). In the enhancing tumor area, NODDI-derived parameters showed either no significant differences (AUC 0.54-0.72), and MAP-MRI parameters reached an AUC of only 0.74, with no metric consistently outperforming conventional diffusion indices; moreover, peritumoral edema showed no significant differences with any diffusion parameter in these studies, and sample sizes were relatively small. Future studies may combine multiple imaging techniques to achieve improved diagnostic accuracy. Habitat imaging based on multimodal MRI can provide deeper insights into intratumoral heterogeneity (26, 27), offering not only conventional information but also revealing the complexity of the tumor microenvironment, thus holding broad application prospects.

This study has several limitations. First, it is a single-center retrospective investigation without external validation, and the sample size is relatively limited; therefore, future multicenter studies with larger cohorts are essential to validate the robustness of the proposed model. Second, the pathological subtypes of solitary brain metastases included in this study were heterogeneous, encompassing primary malignancies of pulmonary, gastrointestinal, and ovarian origin, among others, which may introduce potential bias. Third, the nomogram was developed using data acquired from a single MRI scanner and relied on a specific processing pipeline. This may lead to overfitting and limit generalizability to other imaging protocols or software environments.

In conclusion, hemodynamic parameters and *V*_(IPE)_/*V*_(P)_ derived from tumor vascular habitats can effectively differentiate GBM from SBM. The nomogram model provides a novel non-invasive approach for preoperative differential diagnosis and offers a basis for treatment planning.

## Data Availability

The raw data supporting the conclusions of this article will be made available by the authors, without undue reservation.
