# Supplementary material for: Vascular habitat analysis using dynamic susceptibility contrast-enhanced perfusion MRI for differentiating glioblastoma from solitary brain metastases
Source: Front Oncol. 2026 Oct 7;16:1904498. doi: 10.3389/fonc.2026.1904498 (PMC13683008; doi:10.3389/fonc.2026.1904498)
Supplement: Supplementary file 1 [file Table1.docx]

Supplementary Material

# 1 Supplementary Tables

Table 1 MRI Equipment scanning parameters

| Scanning Sequence | Repetition Time (ms) | Echo Time (ms) | Inversion Time (ms) | Slice Thickness (mm) | Matrix | Field of View (mm) | Flip Angle |
| --- | --- | --- | --- | --- | --- | --- | --- |
| T1WI | 268 | 2.67 | - | 5 | 320×256 | 230×230 | 90° |
| T2WI | 3800 | 96 | - | 5 | 320×320 | 240×240 | 150° |
| FLAIR | 8000 | 94 | 2370 | 5 | 256×256 | 230×230 | 150° |
| CE-T1WI | 250 | 2.67 | - | 5 | 320×256 | 230×230 | 90° |
| DSC-PWI | 1500 | 30 | - | 5 | 128×128 | 240×240 | 60° |

Table 2 Correlation analysis of various habitat hemodynamic parameters between the GBM and SBM groups

| Habitat hemodynamic parameters | Correlation coefficient (r) of GBM group | Correlation coefficient (r) of SBM group |
| --- | --- | --- |
| HAT-rCBV_median_/LAT-rCBV_median_ | 0.744^**^ | 0.797^**^ |
| HAT-rCBV_median_/IPE-rCBV_median_ | 0.559^**^ | 0.070 |
| HAT-rCBV_median_/VPE-rCBV_median_ | 0.283^*^ | 0.032 |
| LAT-rCBV_median_/IPE-rCBV_median_ | 0.576^**^ | 0.230 |
| LAT-rCBV_median_/VPE-rCBV_median_ | 0.362^**^ | 0.286^*^ |
| IPE-rCBV_median_/VPE-rCBV_median_ | 0.685^**^ | 0.683^**^ |
| HAT-rCBF_median_/LAT-rCBF_median_ | 0.764^**^ | 0.688^**^ |
| HAT-rCBF_median_/IPE-rCBF_median_ | 0.546^**^ | 0.101 |
| HAT-rCBF_median_/VPE-rCBF_median_ | 0.314^**^ | 0.053 |
| LAT-rCBF_median_/IPE-rCBF_median_ | 0.570^**^ | 0.294^*^ |
| LAT-rCBF_median_/VPE-rCBF_median_ | 0.408^**^ | 0.299^*^ |
| IPE-rCBF_median_/VPE-rCBF_median_ | 0.790^**^ | 0.799^**^ |

Note: *and**indicate a significant correlation at the level of *P*<0.05 and a highly significant correlation at the level of *P*<0.01(two-sided), respectively.
